# The humanistic and economic burden of work-related musculoskeletal pain: a cross-sectional survey of workers in the United Kingdom

**DOI:** 10.1186/s13104-023-06461-5

**Published:** 2023-08-24

**Authors:** Adam B. Smith, Stephanie Cooper, Jennifer Hanning, Carolyn Buckley

**Affiliations:** 1 Health Outcomes, Reckitt, Slough, UK; 2 Global Medical Affairs, Reckitt, Slough, UK

**Keywords:** Work-related musculoskeletal pain, Lower body pain, Health-related quality of life, Economic cost, Absenteeism, Presenteeism, Work productivity, Questionnaire

## Abstract

**Objective:**

The aim of this study was to evaluate the impact of work-related musculoskeletal (MSK) lower body pain on health-related quality of life (HRQoL) and work productivity in a large sample of workers in the United Kingdom, as well as evaluating the potential economic impact of MSK pain.

**Methods:**

Participants with self-reported work-related MSK pain were recruited from an online panel maintained by a third party (Qualtrics LLC). Participants completed three validated instruments online: the Brief Pain Inventory (BPI), the Assessment of Quality of Life Instrument (AQoL-4D), and the 6-item Work Productivity and Activity Impairment Questionnaire (WPAI). Sociodemographic details, work patterns and healthcare resource utilisation were also reported. One-way analysis of variance (ANOVA) and t-tests were used to explore differences between variables. Linear regression was applied to determine the impact of work-related MSK pain on HRQoL.

**Results:**

All 1035 recruited participants completed the survey (57.4% female; mean age 43.4 years). Participants reported spending all (25.2%) or most (53%) of their time at work on their feet. Mean pain severity was 4.63 (standard deviation: 2.07); mean pain interference was 4.37 (2.49). There was a linear relationship between length of shift, time on feet and pain. Mean AQoL-4D scores were 0.609 (0.254). A mean of 4.12 h was lost per week due to pain. Absenteeism (last 7 days) was 9.5% (20.7%), and presenteeism 33.3% (24.9%). An average 1.55 visits were made to family practitioners (total cost: £19,866) and 1 hospital visit (£37,320) due to work-related MSK pain.

**Conclusion:**

This study demonstrated that work-related lower body pain has a significant impact in terms of individual HRQoL and as an economic societal burden.

## Introduction

A significant number of people are standing throughout the course of the day because of their occupation. In the European Union (EU), almost two thirds (62%) of the working population stand for most of their working day [1]. In the United States (US), over half of the adults surveyed (52%) reported that they experience tired, sore feet during or after work [2], and a recent review in the United Kingdom (UK) found that at least half the working population experience prolonged standing, associated with a negative impact on the body and a high prevalence of musculoskeletal (MSK) disorders of the feet, lower extremities and lower back [3–8].

In occupations such as factory workers [9], laboratory workers [10], postal workers [11], healthcare workers [5] or those in the police force [4], around 20% of those who spend most of their working day on their feet experience foot pain or discomfort [4].

Although previous studies have explored the impact of work-related pain on productivity, particularly presenteeism, these have predominantly focused on lower back pain or on workers with diagnosed medical conditions [12, 13]. To the authors knowledge there has been no focus to-date on the impact of lower-body work-related pain on health-related quality of life (HRQoL) in general, and specifically on work productivity (in the UK). Therefore, this study aimed to evaluate the impact of work-related musculoskeletal (MSK) lower body pain on health-related quality of life (HRQoL) and work productivity in a large sample of workers in the United Kingdom, as well as evaluating the potential economic impact of MSK pain.

## Methods

### Survey

The study design was a cross-sectional survey using self-selection sampling. The survey was conducted in accordance with the principles outlined in the Declaration of Helsinki. Participants were presented with the survey background, including the study purpose, questions, and sponsor (Reckitt). Subsequently, participants were asked to provide their consent to participate and then directed to the survey. Participants were informed that they were able and entitled to leave the survey at any time, and that any data collected up to that point would not be stored nor utilised in the data analysis. Data were collected on work-related MSK pain, sociodemographics and health. This study comprised two samples: one collected entirely in Scotland (April to May 2018); the other sample was designed to be representative of the UK general population as a whole (October to November 2018).

### Participants

The target sample consisted of adults who met the following inclusion criteria: aged 18–67 years, from the UK population, with self-reported lower body musculoskeletal pain either as a long-term or transient condition resulting from having to be stood as part of their employment. Participants not meeting these screening criteria were excluded from the survey. Participants were drawn from an online panel maintained by a third party (Qualtrics LLC). Potential participants were invited to participate by Qualtrics. Data collected by Qualtrics were anonymised before being shared with the research team to ensure no personally identifiable information was provided to the latter.

Although no formal sample size calculations were undertaken, a sample size in excess of N = 1000 was deemed sufficient to provide a representative sample of the UK population for descriptive purposes and exploratory analyses.

### Instruments

Participants completed three validated instruments. The 9-item Brief Pain Inventory (BPI) [14] was used capture the severity of pain and pain interference. The 6-item Work Productivity and Activity Impairment Questionnaire (WPAI) [15] measured the impact of work-related MSK pain on respondents’ absenteeism, presenteeism, activity impairment and overall impairment. The 12-item Assessment of Quality of Life Instrument (AQoL-4D) [16] was used to assess HRQoL and to provide a measure of independent living, relationships, mental health and the senses.

#### Economic costs

The estimated cost of presenteeism was derived using industry-specific weekly median wages for the United Kingdom (UK). This was calculated using duration time the participant had been experiencing work-related MSK pain multiplied by the individual level of presenteeism and weekly median wage. Duration of time pain had been experienced was captured on a 12-point scale: less than 1 month, individual months from 1 to 10, and more than 10 months. For this calculation, less and 1 month and 1 month were combined, and > 10 months interpreted as 12 months in order to convert the time period into one year. Participants were not asked to provide an indication of presenteeism due to work-related pain beyond the 7 days recorded in the WPAI. This weekly estimate of presenteeism was assumed to hold across the length of time participants indicated to have been affected by work-related pain. This therefore provides a conservative estimate of the impact of pain on presenteeism across the year.

Unit costs for family practitioner consultations for 2019–2020 were £33 per consultation lasting 9.22 min (including direct care) [17]. The average outpatient consultation was estimated to be £120 [18]. These figures were used with participants’ self-reported attendance at GP and hospital consultations to provide estimates of the direct costs to the UK National Health Service (NHS).

#### Statistics

Descriptive statistics were derived from the participants’ responses (mean and standard deviation for quantitative data, medians and range for qualitative data). One-way analysis of variance (ANOVA) was used to explore differences between groups of variables. Linear regression was applied to determine the impact of work-related MSK pain on HRQoL controlling for age, gender, absenteeism, presenteeism, length of time standing at work and general health. The data were analysed using SAS [19] version 9.4.

## Results

### Sample characteristics

A total of 1035 participants took part in the study (100% of those recruited): 57.4% females (594) and 42.5% males (440). Average age was 43.35 years (18 to 67 years). Participants mostly rated their general health as either average (42.4%) or good (35.6%). The most frequent sectors of occupation were healthcare (17.3%), hospitality and associated industries (12.5%), teaching and education (11.5%) and sales (11.0%) (Table 1).

**Table 1:** Distribution of occupations

Sector	N	%
**Healthcare**	179	17.3
**Hospitality, leisure & travel**	129	12.5
**Teaching & education**	118	11.4
**Sales**	114	11.0
**Public service, charity & social work**	92	8.9
**Construction & building services**	70	6.8
**Engineering**	66	6.4
**Logistics, transport & supply chain**	65	6.3
**Consumer goods & FMCG**	39	3.8
**IT & technology**	33	3.2
**Financial services & insurance**	31	3.0
**Science & research**	18	1.7
**Management, business & consulting**	16	1.5
**Accountancy & financial management**	12	1.2
** h & recruitment**	12	1.2
**Law**	11	1.1
**Media, journalism & publishing**	8	0.8
**Property**	7	0.7
**Civil and structural engineering**	5	0.5
**Marketing, advertising & PR**	5	0.5
**Investment banking & investment**	4	0.4

Data on length of daily shifts were collected on 510 participants. The majority of these shifts were ≥ 8 h (60.8%); 22.9% were 6–7 h and 16.4% of shifts were 5 h long; Around 78% of participants were on their feet during a shift either all (25.2%) or most of the time (53%).

### Self-reported pain

The majority of participants did not have any pre-existing medical condition (N = 798, 74.6%). Of those who did (N = 237, 22.9%) the most commonly reported condition was arthritis (19.8%), followed by plantar fasciitis (14.1%) and disc problems (10.3%).

The length of time participants had been experiencing work-related MSK pain was recorded for 510 individuals. Of these 399 (78.2% had no pre-existing medical condition); 57.4% (299) of participants had experienced work-related pain for more than 10 months and only around 9% (28) had experienced pain for 1 month or less. Pain was most frequently experienced after each shift (27.1%) or in the evening (15%). Pain was also experienced in the morning (8.2%), and during the shifts (7.7%).

### Brief Pain Inventory (BPI)

The mean BPI-Severity score was 4.63 (SD: 2.07) and 4.37 (SD: 2.49) for the BPI Interference domain (Table 2). Mean BPI-Severity and Interference were higher for those participants with pre-existing conditions. These differences were statistically significant. The impact of working patterns and pain was explored further for those participants with no pre-existing medical condition (Table 2). A clear gradient was shown for both the time spent standing during a shift as well as the length of time of a shift and pain, with both pain severity and interference increasing relative to the length of time spent standing and duration of the shift. These differences were not statistically significant.

**Table 2:** Mean BPI scores by pre-existing condition, time on feet and daily shift patterns

Pre-existing condition	BPI Severity [mean (SD)]	BPI Interference [mean (SD)]
No (N = 798)	4.45 (2.06)	4.14 (2.45)
Yes (N = 237)	5.23 (2.03)	5.17 (2.48)
	t(1,1033) = 680.2, p < 0.0001	t(1,1033) = 1032.3, p < 0.0001
**Time on feet (N = 797)**		
Little time	4.00 (2.09)	3.25 (1.63)
Half of the time	4.21 (1.96)	3.86 (2.26)
Most of the time	4.42 (1.96)	4.11 (2.44)
All of the time	4.71 (2.29)	4.42 (2.62)
	F(3,793) = 1.95, p = 0.12	F(3,793) = 1.78, p = 0.15
**Daily shift (N = 398)**		
5 h per day	4.59 (2.12)	4.41 (2.55)
6 h per day	4.64 (2.11)	4.16 (2.94)
7 h per day	4.72 (1.92)	4.44 (2.25)
8 h per day	4.84 (1.95)	4.64 (2.33)
More than 8 h per day	5.05 (2.22)	5.03 (2.66)
	F(4,393) = 0.63, p = 0.64	F(4,393) = 1.18, p = 0.32

*SD: standard deviation; F: F-ratio; t: t-value

### AQoL-5D

The mean quality of life score on the AQoL-5D was 0.609 (N = 508) (SD: 0.254, range − 0.04 to 1.00). Differences between participants with and without a pre-existing medical condition were observed in all AQoL-4D domains including overall quality of life (Table 3). These were statistically significant except for the Senses domain.

**Table 3:** AQoL-4D scores by pre-existing condition

Mean (SD)	Independent Living	Relationships	Senses	Mental Health	Quality of life
No pre-existing condition (N = 397)	0.9183 (0.156)	0.8271 (0.2108)	0.9354 (0.0996)	0.8262 (0.1413)	0.6302 (0.2484)
Pre-existing condition (N = 111)	0.8527 (0.1975)	0.7941 (0.2305)	0.9099 (0.1341)	0.7561 (0.1759)	0.531 (0.2586)
	t(1,506) = 182.3, p < 0.0001	t(1,506) = 4.2, p = 0.15	t(1,506) = 23.4,p = 0.03	t(1,506) = 362.1,p < 0.0001	t(1,506) = 184.7,p < 0.0001

*SD: standard deviation; F: F-ratio; t: t-value

### Work productivity - WPAI

The average number of hours worked (N = 934) in the preceding 7 days was 32.38 h (SD: 13.94 h). An average of 4.12 h (SD: 9.73 h) was lost due to work-related pain (WPAI 2) amounting to just under 10% of absenteeism (SD: 20.7%). Presenteeism whilst at work was 33.3% (SD: 24.9%). Percentage overall work impairment and activity impairment were 37.3% (SD: 27.9%) and 36.9% (SD: 25.5%) respectively. Participants with a pre-existing medical condition were more negatively affected in terms of all domains on the WPAI (Table 4). These differences were statistically significant except for Absenteeism.

**Table 4:** Work Productivity

Percentage (SD)	Absenteeism	Presenteeism	Overall Impairment	Activity Impairment
No pre-existing condition (N = 724)	9.05 (20.17)	31.58 (24.45)	35.68 (27.71)	34.6 (24.85)
Pre-existing condition (N = 210)	11.08 (22.46)	39.10 (25.66)	42.86 (27.96)	44.67 (26.82)
	F(1,932) = 1.55,p = 0.21	F(1,932) = 15.07,p < 0.0001	F(1,932) = 10.90,p = 0.001	F(1,932) = 25.78,p < 0.0001

*SD: standard deviation; F: F-ratio

### The relationship between quality-of-life, pain, general health and work productivity

The multivariate linear regression (no pre-existing medical condition, N = 396) (R^2^ = 0.58) showed that both BPI-Interference (standardised beta (b)= -0.021, t=-4.67, p < 0.0001) and Presenteeism (b = -0.132, t=-2.34, p = 0.02) had a negative, statistically significant effect on HRQoL. General health had a positive relationship with HRQoL (b = 0.234, t = 5.44, p < 0.0001). No other variables were statistically significant.

### The economic impact of presenteeism and NHS costs

#### Presenteeism costs

Total cost estimates are provided in Table 5 by industry (no pre-existing medical condition, N = 397). The estimated total weekly cost of presenteeism (industries where N ≥ 10) was £75,444 and the annual cost £640,276. The industries most affected were Sales, HR and Recruitment, Public Sector and Construction.

**Table 5:** Estimated cost of presenteeism

Employment	N	Percent	Weekly wage (£)	Presenteeism Weekly Cost (£)	Presenteeism Annual Cost (£)
**HR & recruitment**	70	17.6	474	10,464	98,160
**Sales**	40	10.1	861	13,259	108,486
**Teaching & education**	40	10.1	490	4459	43,169
**Healthcare**	39	9.8	480	5359	44,643
**Public service, charity & social work**	34	8.6	611	6171	63,300
**Construction & building services**	31	7.8	687	7557	65,815
**Engineering**	30	7.6	721	6129	50,326
**Logistics, transport & supply chain**	28	7.1	651	4883	40,297
**Investment banking & investment**	18	4.5	1321	5358	47,846
**Consumer goods & FMCG**	16	4.0	721	2956	21,125
**Financial services & insurance**	15	3.8	1321	6869	51,255
**Science & research**	10	2.5	861	1980	5855
**Accountancy & financial management**	5	1.3	1321	1585	13,738
**Management, business & consulting**	5	1.3	861	947	4305
**Media, journalism & publishing**	4	1.0	861	1205	13,432
**Hospitality, leisure & travel**	3	0.8	466	379	2560
**Marketing, advertising & PR**	3	0.8	861	689	6544
**Property**	3	0.8	594	356	3802
**IT & technology**	2	0.5	940	1717	7926
**Law**	1	0.3	861	517	6199
**Total**	397			£82,840	£698,781
**Total excl. sectors where N < 10**	371			£75,444	£640,276

### NHS costs

The number of GP and hospital visits made over the last 12 months for work-related MSK pain were recorded by 510 participants. There were 399 participants with no pre-existing medical conditions: 388 (97.2%) participants had visited their GP over this time (total: 602); 395 (99%) had attended an outpatient appointment (total: 311). The average GP appointments per participant was 1.55 with 0.79 outpatient appointments. The estimated total cost for GP appointments was £19,866; and £37,320 for outpatient appointments (average £51.20 and £94.49 per patient respectively).

## Discussion

The aim of this study was to determine the degree of musculoskeletal pain experienced by workers who through their occupation have to spend significant time standing or walking, as well as to explore the relationship between work-related pain, HRQoL and work productivity.

The results demonstrated that being on your feet for a large part of individual workers’ shifts leads to MSK pain. Pain was most frequently experienced after each shift or in the evening with time spent on feet and length of the shift being strongly associated with pain severity (whether that be in workers with diagnosed medical conditions or those non-diagnosed), although this was not statistically significant. These results are in line with foot pain or discomfort reported by those working in a range of occupations requiring prolonged standing, including factory workers [9], laboratory workers [10], postal workers [11], healthcare workers [5] or those in the police force [4].

In terms of pain severity, despite not having ‘diagnosed pain’, this population of workers experienced similar pain severity compared with those who have a pre-existing condition. Therefore, non-diagnosed work-related MSK pain is also significant in a population who spend the majority of their working day on their feet - even more so, when considering that almost two-thirds of this sample had been experiencing pain for close to a year. The levels of pain reported by participants on the BPI is comparable to similar studies that have investigated, for instance, foot joint pain in workers [13], and exceeds levels reported for office workers with lower back pain [20].

The vast majority of participants had visited their family practitioner and/or attended an outpatient appointment for work-related MSK pain. Although this represented a significant cost and time burden to family practitioners with total visits amounting to 90 h of GP time, a *post hoc* analysis of the whole sample (BPI Question7) demonstrated that of those participants with no pre-existing (or diagnosed) condition only 5.3% (N = 42) reported having prescription medicine for their pain. Interesting, only just over a third of those participants (37%) reported at least some pain relief from their (predominantly over-the-counter) medication with less than 1% reporting complete pain relief (BPI Question 8). Given the levels of pain reported by these workers and the length of time that many of them have been experiencing pain in the absence of a diagnosis, it may well be that occupational MSK pain is being underdiagnosed in this population.

Additionally, pain was shown to negatively impact participants’ HRQoL: even those without pre-existing medical conditions were reporting lower HRQoL scores (0.61) compared to general population norms (0.81) [21]. Furthermore, both level of pain and HRQoL were shown to detrimentally impact work productivity. Presenteeism was particularly affected, with an average 13 h per working week (assuming a standard working week of 40 h) where participants may not have been fully productive owing to their work-related MSK. The impact on work productivity was also shown to have a potentially significant negative financial cost to employers.

Given the prevalence of work-related MSK and the wide variety of sectors impacted, better occupational screening and interventions are required to mitigate the cost to the individual in terms of presenteeism, wages and HRQOL, as well as to employers and health services.

### Limitations

There are some potential limitations:

- Work productivity and pre-existing medical conditions were self-reported;
- The economic costs were estimated from UK median salaries, rather than individually reported loss of earnings.

Nevertheless, the large sample size (N > 1000) should mitigate against these limitations.

## Conclusion

Occupation-related body pain was shown to have a significant detrimental impact on individual workers’ health-related quality of life and work productivity across a wide range of industries in the UK. These results demonstrate the humanistic burden of work-related lower body MSK pain, particularly undiagnosed pain on workers, as well as the broader economic impact on employers and society.

## Data Availability

The datasets generated during and analysed during the current study are available from the corresponding author on reasonable request.
